# Hyperglycemia raises the threshold of levosimendan- but not milrinone-induced postconditioning in rat hearts

**DOI:** 10.1186/1475-2840-11-4

**Published:** 2012-01-12

**Authors:** Shuhei Matsumoto, Sungsam Cho, Shinya Tosaka, Ushio Higashijima, Takuji Maekawa, Tetsuya Hara, Koji Sumikawa

**Affiliations:** 1 Department of Anesthesiology, Nagasaki University School of Medicine, Nagasaki, Japan; 2 National Hospital Organization Nagasaki Medical Center, Nagasaki, Japan

**Keywords:** Hyperglycemia, Postconditioning, Myocardial Infarction, Milrinone, Levosimendan, Mitochondrial permeability transition pore

## Abstract

**Background:**

The authors examined whether milrinone and levosimendan could exert cardiac postconditioning effects in rats under normoglycemia and hyperglycemia, and whether the effects could be mediated by mitochondrial permeability transition pore (mPTP).

**Methods:**

Wistar rats underwent 30-min coronary artery occlusion followed by 2-h reperfusion. The rats received milrinone or levosimendan just before reperfusion under normoglycemic or hyperglycemic conditions with or without atractyloside, an mPTP opener.

**Results:**

Under normoglycemia, both 30 μg/kg milrinone (29 ± 12%) and 10 μg/kg levosimendan (33 ± 13%) reduced infarct size compared with that in the control (58 ± 7%). Under hyperglycemia, milrinone (34 ± 13%) reduced infarct size at the same dose as under normoglycemia. In contrast, neither 10 nor 30 μg/kg levosimendan protected hyperglycemic hearts, and only 100 μg/kg levosimendan (32 ± 9%) reduced infarct size compared with that in the hyperglycemic control (58 ± 13%). All of these cardioprotective effects under normoglycemia and hyperglycemia are abolished by atractyloside.

**Conclusion:**

Milrinone and levosimendan exert postconditioning effects via inhibition of mPTP opening. Hyperglycemia raises the threshold of levosimendan-induced postconditioning, while milrinone-induced postconditioning is not influenced by hyperglycemia.

## Introduction

Hyperglycemia (HG) is frequently observed in patients with acute myocardial ischemic events such as myocardial infarction or the usage of cardiopulmonary bypass in cardiac surgery. Many studies have demonstrated the association between HG and increased mortality after acute myocardial infarction [1]. One possible cause of high mortality may be poor cardioprotective strategies under HG. HG was shown to abolish cardioprotection induced by ischemic and pharmacological preconditioning (PreC) [2,3]. Postconditioning (PostC) is more likely than PreC to be feasible as a clinical application, and may be useful in unpredictable myocardial ischemia-reperfusion injury. It was also reported that HG abolished both ischemic and pharmacological PostC [4-6]. To save the ischemic myocardium exposed to HG environment, it is essential to investigate the effective PostC maneuver under HG.

Milrinone, a phosphodiesterase 3 inhibitor (PDE-I), and levosimendan, a calcium sensitizer, are relatively new types of inotropic agent and known to facilitate functional recovery from myocardial ischemia-reperfusion injury (e.g., cardiac surgery under cardiopulmonary bypass and conditions after cardiopulmonary resuscitation), and could lead to preserved perfusion of major organs after myocardial reperfusion [7-10]. At the cardiomyocyte level, intracellular calcium overload occurs during post-ischemic reperfusion, and could cause cardiac arrhythmias or myocardial stunning. PDE-I causes the activation of cyclic adenosine monophosphate (cAMP) and protein kinase A (PKA), resulting in altered calcium handling by sarcoplasmic reticulum (SR) [11]. Levosimendan improves cardiac contractility without change in intracellular calcium [12]. Thus, the properties of these drugs are favorable for myocardial post-ischemic reperfusion period.

Levosimendan has a vasodilatory effect via opening of ATP-sensitive potassium (K_ATP_) channels in the plasma membrane of vascular smooth muscle cells, and also activates myocardial mitochondrial K_ATP _(m-K_ATP_) channels and exerts PreC effect against ischemia-reperfusion injury [13]. Besides PreC, PostC induced by levosimendan has been reported in recent years and it has been suggested that m-K_ATP _channels and phosphatidylinositol 3-kinase (PI3K) are involved in the mechanisms [14,15]. PDE-Is also has PreC properties via activation of the cAMP/PKA pathway independent of protein kinase C (PKC) and m-K_ATP _channels [16,17]. Recently, it was shown that milrinone and levosimendan treatment started prior to reperfusion exerted effects to limit right ventricular infarct size [18].

HG normally elevates intracellular ATP, and m-K_ATP _channel opening-dependent cardioprotective effects would be abolished under HG [19]. Kehl et al. [3] showed that moderate hyperglycemia (blood glucose level of 300 mg/dl) blocked the protective effects of 0.5 minimum alveolar concentration (MAC) but not 1.0 MAC isoflurane. Tsang et al. [20] showed that three-cycle but not one-cycle ischemic PreC reduced myocardial infarct size, and stated that it seems necessary to increase the ischemic PreC stimulus to achieve the threshold for cardioprotection against diabetic myocardium. PDE-I-induced PostC, which is independent of m-K_ATP _channels, would not be impaired by HG.

Multiple lines of evidence suggest that the mitochondrial permeability transition pore (mPTP) is a key end effector of ischemic and pharmacological PostC [21], and the important link between Reperfusion Injury Salvage Kinases (RISK)/Survivor Activating Factor Enhancement (SAFE) pathways and mPTP has also been suggested [21,22]. Under HG, adequate mPTP closing maneuver would be essential for myocardial infarct size reduction [5].

In the present study, we hypothesized that HG would raise the threshold of levosimendan-induced PostC, which is mediated by m-K_ATP _channels, and we also examined whether milrinone- and levosimendan-induced PostC could be mediated by the inhibition of mPTP opening.

## Materials and methods

All experimental procedures and protocols described in this study were approved by the Institutional Animal Care and Use Committee of the Nagasaki University School of Medicine.

### Drugs

Milrinone was purchased from Astellas Pharma Co. (Tokyo, Japan). Levosimendan, atractyloside, and 2,3,5-triphenyltetrazolium chloride (TTC) were purchased from Sigma (St. Louis, MO, USA).

### General Preparation

The instrumental methods used were as described in our previous report [17]. Male Wistar rats weighing between 250 and 450 g were anesthetized with sodium pentobarbital (a 50 mg/kg intraperitoneal bolus followed by an intravenous infusion at 10-20 mg/kg/h). The rats were adequately sedated to ensure that pedal and palpebral reflexes were absent throughout the experimental protocol. Catheters were inserted into the right jugular vein and the right carotid artery for fluid or drug administration and measurement of arterial blood pressure, respectively. After tracheotomy, the trachea was intubated with a cannula connected to a small animal ventilator (model SAR-830 CWE, PA, USA), and the lungs were ventilated with pure oxygen. Arterial blood gas pH was maintained within a physiological range by adjusting the respiratory rate and tidal volume throughout the experiment. A left thoracotomy was performed in the fifth intercostal space, and the pericardium was opened. A 7-0 prolene ligature was placed around the proximal left anterior descending coronary artery (LAD) and vein in the area immediately below the left atrial appendage. The ends of the suture were threaded through a small plastic tube to form a snare for reversible LAD occlusion. Coronary artery occlusion was produced by clamping the snare onto the epicardial surface of the heart and was confirmed by the appearance of epicardial cyanosis. Reperfusion was achieved by loosening the snare and was verified by observing an epicardial hyperemic response. Hemodynamics were continuously monitored with a transducer (blood pressure monitor link sck-9082; Becton Dickinson, Tokyo, Japan) and an AP-641G blood pressure amplifier (Nihon-Kohden, Tokyo, Japan) and shown on a polygraph system (Nihon-Kohden).

### Experimental Protocol

**Figure 1:**
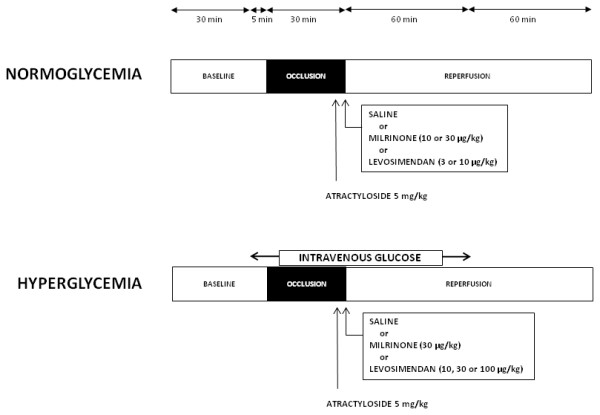
**A schematic illustration of the experimental protocols**.

The experimental design used in the current investigation is illustrated in Figure 1. All rats underwent 30-min coronary artery occlusion followed by 2-h reperfusion.

### Protocol 1: The threshold of milrinone- and levosimendan-induced PostC under normoglycemia (NG)

The experiments were performed under NG. Wistar rats were allocated to one of 5 groups. The rats received saline (group CON; n = 14), milrinone at 10 μg/kg (group MIL10; n = 8), milrinone at 30 μg/kg (group MIL30; n = 9), levosimendan at 3 μg/kg (group LEV3; n = 7), or levosimendan at 10 μg/kg (group LEV10; n = 9). These drugs were administered as i.v. bolus just prior to reperfusion.

### Protocol 2: The threshold of milrinone- and levosimendan-induced PostC under HG

The experiments were performed under HG. Wistar rats were allocated to one of 5 groups. To produce HG, rats received 50% glucose intravenously starting 5 min before ischemia and lasting until 60 min after reperfusion. The target blood glucose level was 300 mg/dl throughout the experiment. To test the drugs, the rats received saline (group HG CON; n = 10), milrinone at 30 μg/kg (group HG MIL30; n = 7), levosimendan at 10 μg/kg (group HG LEV10; n = 9), levosimendan at 30 μg/kg (group HG LEV30; n = 7), or levosimendan at 100 μg/kg (group HG LEV100; n = 7) just prior to reperfusion. The doses were set on the basis of the results of protocol 1.

### Protocol 3: The role of mPTP in milrinone- and levosimendan-induced postconditioning

Atractyloside, an mPTP opener (5 mg/kg), was administered intravenously 5 min before reperfusion, and the protective doses of milrinone or levosimendan were administered just before reperfusion under NG and HG. The doses of atractyloside were set on the basis of previous studies [23,24]. The protective doses of test drugs were set on the basis of protocols 1 and 2 as follows: milrinone at 30 μg/kg and levosimendan at 10 μg/kg under NG, and milrinone at 30 μg/kg and levosimendan at 100 μg/kg under HG.

### Blood Glucose Measurement

Blood samples were collected to measure blood glucose in all rats. The blood glucose level was determined by the glucose oxidase method using a Glutest sensor and Glutest Ace (Sanwa Kagaku Kenkyusho, Nagoya, Japan). Samples were taken before starting glucose administration, just before ischemia (after starting glucose administration), just after starting reperfusion, and 1 and 2 hr after reperfusion.

### Determination of Infarct Size

**Figure 2:**
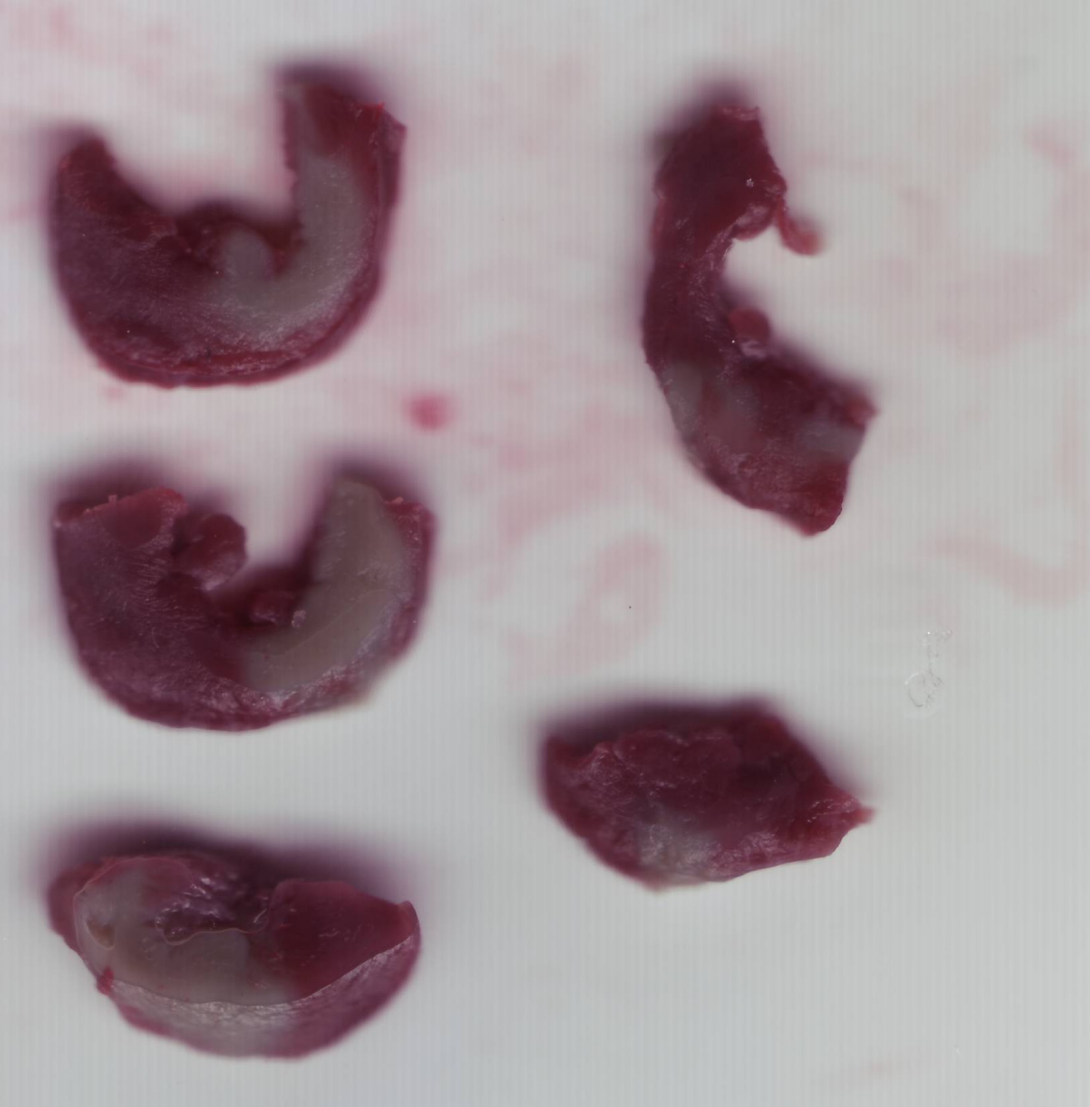
**Left ventricular tissues after 2,3,5-triphenyltetrazolium chloride staining**. Non-ischemic areas have been cut off. Areas at risk are stained red and necrotic areas appear pale.

At the end of the experiment, the LAD was reoccluded. Patent blue dye was administered intravenously to stain the normal region of the left ventricle (LV), and the heart was rapidly excised. Excess LV tissue was removed and cut into approximately 10 cross-sectional slices of equal thickness. The nonstained LV area at risk (AAR) was physically separated from the blue-stained LV normal zone, and incubated at 37°C for 15 min in 1% TTC in 0.1 M phosphate buffer adjusted to pH 7.4. The tissue slices were fixed overnight in 10% formaldehyde. LV tissues after TTC staining are shown in Figure 2. TTC stains living tissue deep red, but necrotic tissue is TTC-negative and appears white. Each slice was scanned at 1200 dpi with a commercial scanner (Canoscan LiDE 60; Canon, Japan), and infarcted and noninfarcted areas were measured by one observer blinded to the rat groups using an image analysis program. Myocardial infarct size is expressed as a percentage of the AAR.

### Statistical Analysis

Statistical analysis of hemodynamic data and blood glucose concentrations within and between groups was performed with analysis of variance for repeated measures followed by Dunnett's test. Inter-group differences in body weight, age, LV weight, AAR weight, the ratio of AAR to LV, and the ratio of infarct size to AAR were analyzed using one-way analysis of variance followed by the Student-Newman-Keuls test. Statistical significance was defined as P < 0.05. All values are expressed as mean ± SD. Statistical analysis was performed using SPSS 15.0 software (SPSS Japan, Tokyo, Japan) or GraphPad Prism 5.0 (GraphPad Software, San Diego, CA).

## Results

There were no significant differences in body weight or age among the groups. One hundred and fifty-one rats were used, with 124 successful myocardial infarct size experiments being achieved. Ten rats were excluded as a result of technical difficulties with the experimental preparation. Malignant ventricular arrhythmias developed in 17 other rats before completion of the experiment. Two in the CON group, 1 in the MIL10 group, 2 in the LEV3 group, 1 in the LEV10 group, 1 in the HG CON group, 2 in the HG MIL30 group, 2 in the HG LEV10 group, 1 in the HG LEV30 group, 1 in the ATR .group, 2 in the MIL30 + ATR group, 1 in the LEV10 + ATR group, and 1 in the HG LEV100 + ATR group; no differences in incidence of arrhythmic events were observed among groups. These rats were excluded from further analysis.

### Hemodynamics

Hemodynamic variables are summarized in Table 1 and 2. No significant differences in mean arterial pressure and heart rate were observed between the experimental groups at baseline. In levosimendan-treated groups, mean arterial pressure showed a tendency to decrease transiently during the early reperfusion period. Heart rate increased in the LEV10 group and HG LEV100 group at 2hr after reperfusion as compared with that in the CON group.

**Table 1:** Mean Arterial Pressure

	Number	Baseline	Preocclusion	Occlusion 30 min		Reperfusion		
					
					5 min	30 min	1 hr	2 hr
MAP (mmHg)								
CON	14	99 ± 24	97 ± 14	93 ± 20	95 ± 26	109 ± 28	107 ± 31	100 ± 36
MIL10	8	91 ± 24	88 ± 26	91 ± 23	84 ± 25	100 ± 26	104 ± 20	107 ± 15
MIL30	9	98 ± 14	83 ± 14	91 ± 16	88 ± 22	103 ± 17	99 ± 30	99 ± 31
LEV3	7	97 ± 20	95 ± 16	84 ± 27	87 ± 12	92 ± 16	99 ± 14	106 ± 14
LEV10	9	90 ± 16	84 ± 13	79 ± 28*	80 ± 23*	89 ± 32	93 ± 26	96 ± 22
HGCON	10	109 ± 21	107 ± 23	93 ± 20	92 ± 23	102 ± 26	110 ± 23	112 ± 20
HG MIL30	7	101 ± 13	102 ± 12	92 ± 13	93 ± 11	101 ± 14	109 ± 18	118 ± 19
HG LEV10	9	101 ± 16	91 ± 9	85 ± 10	84 ± 11	94 ± 15	104 ± 17	108 ± 18
HG LEV30	7	105 ± 21	97 ± 11	96 ± 18	93 ± 15	93 ± 12	106 ± 9	104 ± 18
HG LEV100	7	103 ± 18	109 ± 12	79 ± 12*	81 ± 9*	84 ± 13	96 ± 14	94 ± 21
ATR	6	103 ± 13	96 ± 13	108 ± 11	107 ± 15	113 ± 19	113 ± 16	116 ± 12
MIL30 + ATR	8	101 ± 14	93 ± 21	97 ± 14	97 ± 15	101 ± 15	101 ± 16	102 ± 11
LEV10 + ATR	7	87 ± 15	85 ± 15	97 ± 22	95 ± 17	97 ± 24	98 ± 20	96 ± 18
HG MIL30 + ATR	7	98 ± 20	95 ± 17	98 ± 19	100 ± 20	103 ± 21	103 ± 17	88 ± 17
HG LEV100 + ATR	9	93 ± 14	97 ± 18	89 ± 21	88 ± 17	98 ± 21	103 ± 23	101 ± 25

Data are mean ± SD.

MAP = mean arterial pressure; CON = control; MIL10 or 30 = 10 or 30 μg/kg milrinone; LEV 3, 10, 30 or 100 = 3, 10, 30 or 100 μg/kg levosimendan; HG = hyperglycemia; ATR = atractyloside.

*Significantly (*P *< 0.05) different from baseline.

**Table 2:** Heart Rate

	Number	Baseline	Preocclusion	Occlusion 30 min		Reperfusion		
					
					5 min	30 min	1 hr	2 hr
HR (mmHg)								
CON	14	389 ± 39	383 ± 43	375 ± 46	381 ± 51	376 ± 52	374 ± 56	342 ± 68*
MIL10	8	352 ± 51	354 ± 58	345 ± 68	340 ± 64	338 ± 58	339 ± 70	340 ± 72
MIL30	9	381 ± 66	379 ± 65	362 ± 24	372 ± 41	346 ± 61	349 ± 80	335 ± 91*
LEV3	7	370 ± 20	367 ± 15	339 ± 40	356 ± 31	350 ± 38	340 ± 40	338 ± 52
LEV10	9	408 ± 37	396 ± 28	387 ± 45	390 ± 41	395 ± 42	399 ± 45	405 ± 56†
HGCON	10	394 ± 55	377 ± 64	397 ± 40	390 ± 70	391 ± 67	380 ± 76	371 ± 114
HG MIL30	7	366 ± 44	365 ± 44	377 ± 33	381 ± 36	382 ± 50	363 ± 73	373 ± 82
HG LEV10	9	402 ± 35	389 ± 35	364 ± 18	374 ± 15	367 ± 32	366 ± 49	366 ± 56
HG LEV30	7	372 ± 49	376 ± 47	361 ± 45	371 ± 46	364 ± 38	359 ± 35	348 ± 50
HG LEV100	7	433 ± 24	421 ± 32	400 ± 30	413 ± 29	394 ± 45	390 ± 57	402 ± 29†
ATR	6	379 ± 21	372 ± 15	359 ± 17	367 ± 24	369 ± 29	365 ± 33	347 ± 44
MIL30 + ATR	8	377 ± 50	386 ± 34	363 ± 34	372 ± 37	379 ± 40	358 ± 41	367 ± 35
LEV10 + ATR	7	364 ± 34	382 ± 30	335 ± 34	345 ± 45	339 ± 51	331 ± 43	336 ± 52
HG MIL30 + ATR	7	386 ± 53	394 ± 48	396 ± 38	411 ± 46	404 ± 49	407 ± 48	377 ± 60
HG LEV100 + ATR	9	412 ± 27	411 ± 47	387 ± 32	399 ± 33	379 ± 50	384 ± 54	375 ± 47

Data are mean ± SD.

HR = heart rate; CON = control; MIL10 or 30 = 10 or 30 μg/kg milrinone; LEV 3, 10, 30 or 100 = 3, 10, 30 or 100 μg/kg levosimendan; HG = hyperglycemia; ATR = atractyloside.

*Significantly (*P *< 0.05) different from baseline. †Significantly (P < 0.05) different from CON.

### Infarct Size

**Figure 3:**
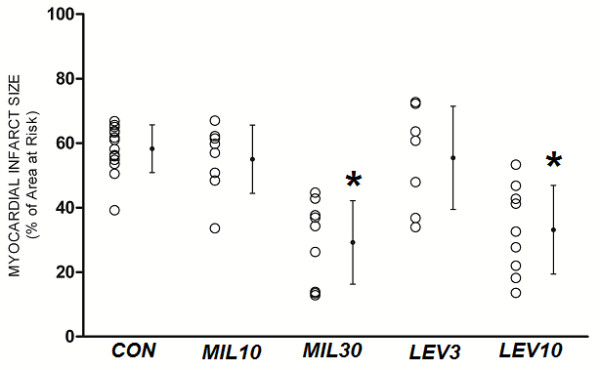
**Myocardial infarct size expressed as a percentage of the left ventricular area at risk in protocol 1**. CON = control; MIL10, 30 = 10 or 30 μg/kg milrinone, LEV3, 10 = 3 or 10 μg/kg levosimendan. Data are mean ± SD. *Significantly (*P *< 0.05) different from CON.

**Figure 4:**
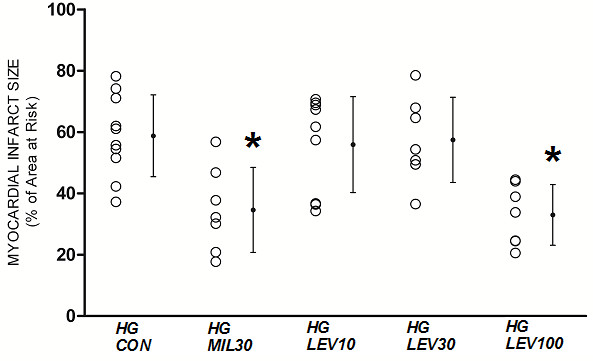
**Myocardial infarct size expressed as a percentage of the left ventricular area at risk in protocol 2**. HG = hyperglycemia; CON = control; MIL30 = 30 μg/kg milrinone, LEV10, 30 or 100 = 10, 30 or 100 μg/kg levosimendan. Data are mean ± SD. *Significantly (*P *< 0.05) different from HG-CON.

**Figure 5:**
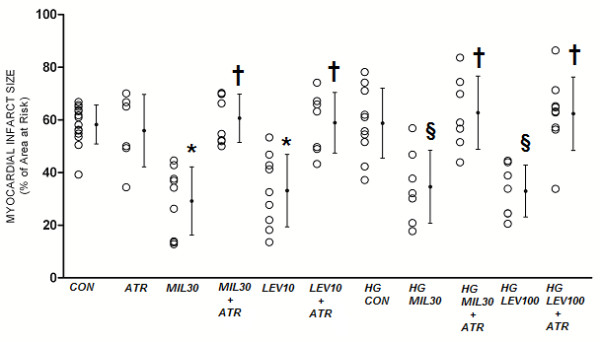
**Myocardial infarct size expressed as a percentage of the left ventricular area at risk in protocol 2**. CON = control; HG = hyperglycemia; ATR = atractyloside; MIL30 = 30 μg/kg milrinone, LEV100 = 100 μg/kg levosimendan. Data are mean ± SD. *Significantly (*P *< 0.05) different from CON. §Significantly (*P *< 0.05) different from HG-CON. †Significantly (*P *< 0.05) different from the group without ATR.

LV weight, AAR weight, and the ratio of AAR to total LV mass were similar among the groups (Table 3). Infarct size expressed as a percentage of area at risk is shown in Figure 3, 4, 5. As shown in Figure 3, in NG rats, 30 μg/kg milrinone and 10 μg/kg levosimendan reduced infarct size compared with that in the control (MIL30: 29 ± 12%; LEV10: 33 ± 13%; CON: 58 ± 7%). In contrast, low-dose groups (10 μg/kg milrinone and 3 μg/kg levosimendan) did not show reduction of infarct size (MIL10: 55 ± 10%; LEV3: 55 ± 16%). As shown in Figure 4, in HG rats, 100 μg/kg but not 10 μg/kg or 30 μg/kg levosimendan reduced infarct size compared with that in the control (HG LEV100: 32 ± 9%; HG LEV10: 55 ± 15%; HG LEV30: 57 ± 13%;HG CON: 58 ± 13%). In contrast, 30 μg/kg milrinone showed similar degrees of infarct size reduction in NG and HG rats (HG MIL30: 34 ± 13%). As shown in Figure 5, atractyloside blocked the beneficial effects of both milrinone and levosimendan (LEV10 + ATR: 58 ± 11%; MIL30 + ATR: 60 ± 9%; HG LEV100 + ATR: 62 ± 13%; HG MIL30 + ATR: 62 ± 13%). Atractyloside alone did not affect infarct size (ATR: 55 ± 13%).

**Table 3:** Left-Ventricular Area at Risk

	Number	Area at Risk/Left Ventricle (%)
CON	14	57 ± 8
MIL10	8	51 ± 9
MIL30	9	52 ± 7
LEV3	7	56 ± 5
LEV10	9	51 ± 5
HG CON	10	50 ± 4
HG MIL30	7	53 ± 3
HG LEV10	9	52 ± 7
HG LEV30	7	56 ± 5
HG LEV100	7	50 ± 10
ATR	6	51 ± 4
MIL30 + ATR	8	48 ± 5
LEV10 + ATR	7	48 ± 8
HG MIL30 + ATR	7	48 ± 8
HG LEV100 + ATR	9	51 ± 4

Data are mean ± SD.

CON = control; MIL10 or 30 = 10 or 30 μg/kg milrinone; LEV 3, 10, 30 or 100 = 3, 10, 30 or 100 μg/kg levosimendan; HG = hyperglycemia; ATR = atractyloside.

### Blood Glucose Measurement

Blood glucose concentrations are summarized in Table 4. No significant differences in blood glucose concentrations were observed between the experimental groups at baseline. After starting administration of 50%-glucose solution, rats in HG groups had significantly higher blood glucose concentrations than rats in NG groups.

**Table 4:** Blood Glucose Concentrations (mg/dl)

	Number	Baseline	Preocclusion	Occlusion 30 min	Reperfusion	
					
					1 hr	2 hr
CON	14	92 ± 23	89 ± 19	109 ± 40	129 ± 43	111 ± 34
MIL10	8	114 ± 75	105 ± 52	132 ± 60	124 ± 56	105 ± 70
MIL30	9	93 ± 23	82 ± 20	107 ± 51	98 ± 46	79 ± 29
LEV3	7	116 ± 40	89 ± 24	138 ± 66	120 ± 80	106 ± 49
LEV10	9	99 ± 39	97 ± 28	122 ± 51	113 ± 33	101 ± 41
HG CON	10	90 ± 16	355 ± 121*^†^	286 ± 137*^†^	361 ± 140*^†^	273 ± 144*^†^
HG MIL30	7	87 ± 16	343 ± 95*^†^	351 ± 62*^†^	295 ± 150*^†^	246 ± 140*^†^
HG LEV10	9	88 ± 17	320 ± 97*^†^	311 ± 120*^†^	321 ± 142*^†^	285 ± 161*^†^
HG LEV30	7	98 ± 32	341 ± 83*^†^	390 ± 99*^†^	313 ± 124*^†^	293 ± 153*^†^
HG LEV100	7	110 ± 45	447 ± 84*^†^	394 ± 113*^†^	408 ± 140*^†^	385 ± 161*^†^
ATR	6	82 ± 38	87 ± 25	93 ± 14	87 ± 25	85 ± 12
MIL30 + ATR	8	80 ± 27	80 ± 15	101 ± 49	90 ± 58	77 ± 47
LEV10 + ATR	7	100 ± 32	100 ± 36	124 ± 57	147 ± 94	123 ± 80
HG MIL30 + ATR	7	74 ± 29*	317 ± 70*^†^	376 ± 31*^†^	293 ± 191*^†^	241 ± 197*^†^
HG LEV100 + ATR	9	76 ± 13	341 ± 145*^†^	379 ± 106*^†^	381 ± 149*^†^	311 ± 131*^†^

Data are mean ± SD.

CON = control; MIL10 or 30 = 10 or 30 μg/kg milrinone; LEV 3, 10, 30 or 100 = 3, 10, 30 or 100 μg/kg levosimendan; HG = hyperglycemia; ATR = atractyloside.

*Significantly (*P *< 0.05) different from baseline. †Significantly (*P *< 0.05) different from CON.

## Discussion

The present study is, to our knowledge, the first to demonstrate that milrinone-induced PostC protects the heart under HG at the same dose as under NG, while levosimendan-induced PostC requires an increased dose under HG. Our results also demonstrate that all of these cardioprotective effects in NG and HG are mediated by mPTP.

### Cardioprotective Signaling Pathways in HG Conditions

It is well known that acute HG condition attenuates endogenous cardioprotective phenomena by ischemic/pharmacological interventions. Ischemic/pharmacological PreC and PostC could inhibit mPTP opening at reperfusion through m-K_ATP _channels and complex signal transduction pathways such as PI3K-Akt, p42/p44 extracellular signal-regulated kinases (ERK 1/2), glycogen synthase kinase-3beta (GSK-3beta), endothelial nitric oxide synthase (eNOS), and PKC [21,22]. Reactive oxygen species (ROS) generated by the mitochondrial electron transport chain during ischemic/pharmacological PreC stimulus have been shown to activate m-K_ATP _channels that confer protection of ischemic myocardium, and the ROS scavengers abolish cardioprotection [25]. Kehl et al. [26] reported that acute HG abolishes isoflurane-induced PreC, but a ROS scavenger, N-acetylcysteine, restores these beneficial effects. They suggested that excessive quantities of ROS generated under HG impair isoflurane-induced activation of m-K_ATP _channels. Raphael et al. [4] showed that acute HG inhibits isoflurane-induced PostC, and stated that this effect seems to be mediated via inhibition of Akt and eNOS activation. Acute HG and diabetes are different pathological conditions. Ku et al. [27] reported that HG-induced ROS generation in cardiomyocytes is linked to diabetic cardiomyopathy through GATA binding protein 4 phosphorylation and a higher expression of cardiac troponin I. Kageyama et al. [28] found that HG up-regulated death receptor expression, coupled with increased tumor necrosis factor (TNF)-alpha secretion, promoted endothelial cell apoptosis, and suggested that it is contributes to coronary arterial endothelial dysfunction and the development of ischemic heart disease in diabetes. Therefore, it is necessary to consider the glycation of intracellular proteins and the effects of complications for patients with diabetes. On the other hand, the inhibitory effects of diabetes on PreC/PostC similar to that of acute HG have also been reported. Tsang et al. [20] showed that three-cycle but not one-cycle ischemia PreC reduced myocardial infarct size in diabetic hearts, commensurate with significant Akt phosphorylation after three cycles of ischemia PreC. Diabetes inhibits erythropoietin- and morphine-induced activation of PI3K-Akt, ERK 1/2, and inhibition of GSK-3beta, and attenuates PostC [29,30]. They also showed that a GSK-3beta inhibitor, SB216763, reduced myocardial infarct size in diabetic rats as well as in non-diabetic rats, and suggested that the mechanism of diabetes-induced attenuation could be due to impairment of protective upstream signaling pathways such as PI3K-Akt and ERK 1/2. Thus poor cardioprotective strategies for both acute HG and diabetes would be derived from the inhibition of the intracellular signaling pathways involved in ischemic and pharmacological PreC and PostC.

### Difference between Protective Effects of Levosimendan and Milrinone under HG

In the present study, levosimendan exerted the PostC effect under HG at a dose ten times higher than the dose under NG. Kersten et al. [19] showed that both 2.5 and 5 μg/kg diazoxide, an m-K_ATP _channel opener, had PreC effects under NG, and that only 5 μg/kg but not 2.5 μg/kg diazoxide reduced myocardial infarct size under acute HG. They suggested that m-K_ATP _channel opening-dependent cardioprotective effects might be attenuated under HG, and thus, increase in the dosage is required. Hönisch et al. [15] showed that levosimendan just prior to reperfusion significantly reduced myocardial infarct size, and this beneficial effect was blocked by 5-hydroxydecanoic acid, an m-K_ATP _channel blocker. Thus, it is likely that HG increases the threshold of levosimendan-induced PostC, resulting from the attenuation of the m-K_ATP _channel opening-dependent cardioprotective effect. In contrast to levosimendan, milrinone-induced PostC protects hearts under HG at the same dose as under NG. Our previous study showed that preischemic administration of olprinone, a PDE-I, reduces myocardial infarct size in type 2 diabetic rats to the same extent as in non-diabetic rats, and that olprinone-induced PreC is independent of m-K_ATP _channels and PKC [17]. Our previous study also showed that levosimendan-induced PostC was dependent on m-K_ATP _channels and PKC; however, milrinone-induced PostC has been suggested to be independent of m-K_ATP _channels and PKC [31,32]. We speculate that milrinone has a different protective pathway leading to mPTP closure independently of m-K_ATP _channels and exerts PostC effects under HG as well as NG.

### Mechanisms leading to mPTP and Cardioprotection

The present results show that levosimendan-induced PostC is mediated by mPTP. Multiple lines of evidence indicate that the mPTP is a key end effector of ischemic and pharmacological PostC [21]. *In vitro *studies using isolated mitochondria have suggested that the opening of m-K_ATP _channels leads to mPTP closure [22]. From this report and our results, the mechanism of levosimendan-induced PostC would involve m-K_ATP _channel and mPTP. The present study also shows that atractyloside, an mPTP opener, prevents milrinone-induced PostC, indicating that this protective effect would be dependent on mPTP. Our previous study showed that preischemic administration of olprinone could confer cardioprotection, and that this cardioprotective effect could be mediated by the activation of PI3K-Akt or by the inhibition of mPTP during early reperfusion [23]. In addition, recent studies have shown that milrinone- and levosimendan-induced PostC increases the level of phosphorylation of Akt [33,34]. The experimental study by Davidson et al. [35] provided evidence that the activation of the PI3K-Akt pathway is linked to the inhibition of mPTP opening. They showed that pharmacological inhibition of either PI3K or Akt abrogated the effect of insulin to prevent mPTP opening in rat cardiomyocytes, and that over-expression of Akt in a cardiac-derived cell line delayed mPTP opening.

### Limitations of the study

In clinical studies, myocardial ischemia is an important prognosis-exacerbating factor in both the diabetic and nondiabetic states of patients who received reperfusion therapy [36]. In addition, adverse events in terms of cardiovascular risk modification are known to occur in both acute and chronic HG [37]. Therefore, the present study was planned and conducted, with a focus on acute HG. However, patients in the clinical setting are in a chronic HG state because of diabetes, making it necessary to consider the glycation of intracellular proteins and the effects of complications such as diabetic cardiomyopathy and endothelial dysfunction. Thus, further studies are needed to apply the results of this experimental study to the clinical setting.

## Conclusions

We have demonstrated that milrinone and levosimendan exert PostC via inhibition of mPTP opening, and that HG raises the threshold of levosimendan-induced PostC, while milrinone-induced PostC is not influenced by HG.

## List of abbreviations

mPTP: mitochondrial permeability transition pore; HG: Hyperglycemia; PreC: preconditioning; PostC: Postconditioning; PDE-I: phosphodiesterase 3 inhibitor; cAMP: cyclic adenosine monophosphate; PKA: protein kinase A; SR: sarcoplasmic reticulum; K_ATP _channels: ATP-sensitive potassium channels; m-K_ATP _channels: mitochondrial ATP-sensitive potassium channels; PI3K: phosphatidylinositol 3-kinase; PKC: protein kinase C; MAC: minimum alveolar concentration; RISK: Reperfusion Injury Salvage Kinases; SAFE: Survivor Activating Factor Enhancement; TTC: 2,3,5-triphenyltetrazolium chloride; LAD: left anterior descending coronary artery; NG: normoglycemia; CON: control; MIL10: milrinone 10 μg/kg; MIL30: milrinone 30 μg/kg; LEV3: levosimendan 3 μg/kg; LEV10: levosimendan 10 μg/kg; LEV100: levosimendan 100 μg/kg; ATR: Atractyloside; LV: left ventricle; AAR: area at risk; SD: standard deviation; ERK 1/2: p42/p44 extracellular signal-regulated kinases; GSK-3beta: glycogen synthase kinase-3beta; eNOS: endothelial nitric oxide synthase; ROS: Reactive oxygen species; TNF: tumor necrosis factor

## Competing interests

The authors declare that they have no competing interests.

## Authors' contributions

SM performed experiments, contributed to discussion, and drafted the manuscript. SC participated in the design and coordination of the study, contributed to discussion and reviewed/edited the manuscript. ST and UH helped carry out in vivo experiments. TM and TH participated in the design and coordination of the study. KS supervised research, contributed to discussion and reviewed/edited the manuscript. All authors have read and approved submission of the final manuscript.
